# Pierre D. and the first photographs of Parkinson's disease

**DOI:** 10.1002/mds.27965

**Published:** 2020-01-24

**Authors:** Patrick A. Lewis, Helene Plun‐Favreau, Mark Rowley, Jennifer Spillane

**Affiliations:** ^1^ School of Pharmacy, University of Reading Whiteknights, Reading United Kingdom; ^2^ Royal Veterinary College London United Kingdom; ^3^ Department of Neurodegenerative Disease UCL Institute of Neurology London United Kingdom; ^4^ Art and Medicine New York New York USA; ^5^ National Hospital for Neurology and Neurosurgery London United Kingdom; ^6^ Guy's and St. Thomas' NHS Foundation Trust, St. Thomas' Hospital London United Kingdom

When William Gowers wrote his *Manual of the Diseases of the Nervous System* in the 1880s, he illustrated the movement disorder Parkinson's disease with an etching of a man displaying the characteristic posture of Parkinson's disease, an etching that was noted as deriving from St. Leger (Fig. 1A).^1^ This image has endured since Gowers’ day as a striking portrayal of Parkinson's disease and reused and adapted in academic publications, lectures, and seminars.

**Figure 1:**
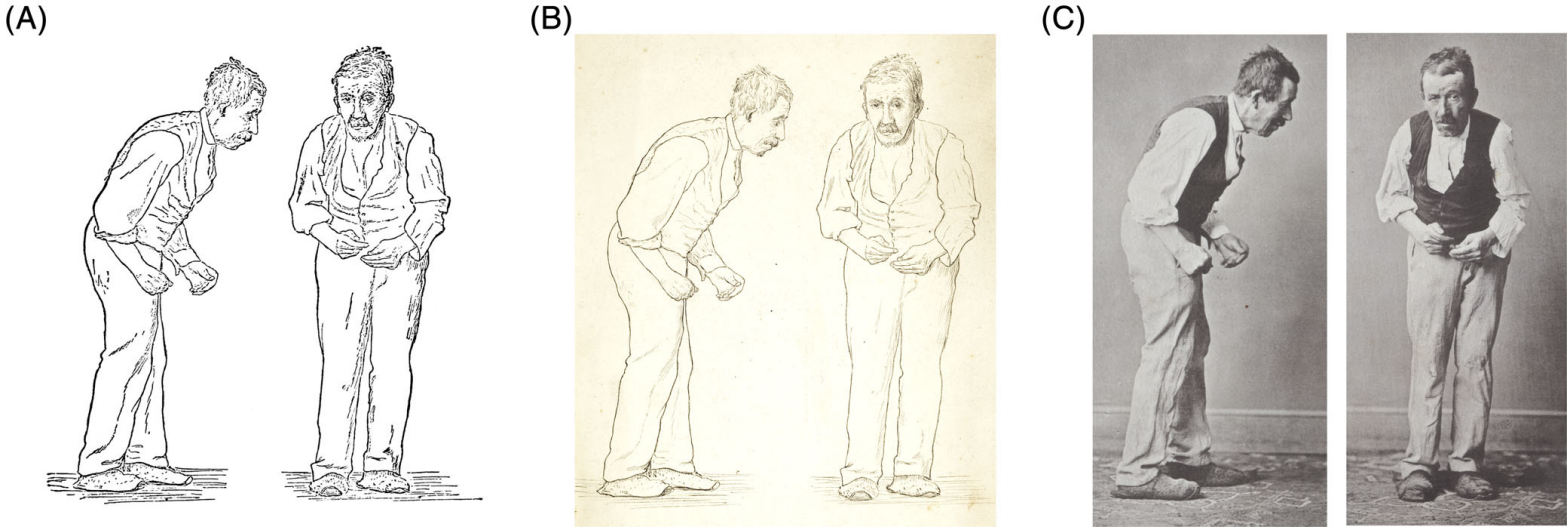
(**A**) The etching of an individual with Parkinson's disease included in William Gowers’ *A Manual of Diseases of the Nervous System*, (**B**) the original drawings by Gowers of this individual, and (**C**) the photos of Pierre D. included in Paul de Saint‐Legers’ thesis on which the Gowers drawing is based. High‐resolution images for (**B**) and (**C**) are available as Supporting Information Files 1 and 2. The image of Gowers’ original drawing was kindly provided by the Queen Square Library archive. [Color figure can be viewed at http://wileyonlinelibrary.com]

The original drawing by Gowers (Fig. 1B) reveals a level of detail sadly lost in the etching and characteristic of an individual who had artistic work exhibited at the Royal Academy of Arts.^2^ This image, held in the archives of the Queen Square Library, was based on an earlier series of photographs of a patient at the Hôpital Salpêtrière (Fig. 1C). These were included in the doctoral thesis of a student of Jean‐Martin Charcot: Paul de Saint‐Léger.^3^ The patient, named in the thesis as Pierre D., was the subject of a detailed case report—one of several included in the thesis. What then do we know of Pierre D.?

The case history included in Saint‐Léger's thesis builds on an earlier description of the patient by another student studying under Charcot, Albert Boucher,^4^ and reports that Pierre was a master mason born between 1822 and 1823 in Aubusson, a town in the Creuse region of central France. He was examined on several occasions during the course of 3 years (in 1876, 1877, and 1879), providing a detailed longitudinal aspect to the case that was lacking from James Parkinson's original description of the shaking palsy. Pierre first noted symptoms in May 1871, putting the age at onset of symptoms before the age of 50. He initially presented with rigidity and tremor upon examination as well as a distinctive posture. Saint‐Léger's report noted a number of striking observations, for example, that Pierre's elbows appeared glued to the body (“*Les coudes sont collés au corps*”), indicative of a reduced arm swing. He also noted that Pierre had a mask‐like, surprised expression and that he walked as though pushed by an invisible hand. Combined with the distinctive posture, this description would in the present day be characterized as parkinsonian. The thesis also reported excessive salivation and a monotonous tone of speech, again signs that are reminiscent of our current characterization of Parkinson's disease. It is noteworthy that a number of these symptoms are apparent in the photographs included in the thesis, described as being provided by M. Landouzy (a reference to the neurologist Louis Landouzy who was studying in Paris at the time).^5^ Saint‐Léger went on to comment that Pierre's stiffness and slowness of movement led him to be nicknamed the “wooden saint” (“*le saint de bois*”) by fellow patients. He provided 2 samples of handwriting (Fig. 2), one from November 1876 (again provided by Dr Landouzy) and one from January 1879.

**Figure 2:**
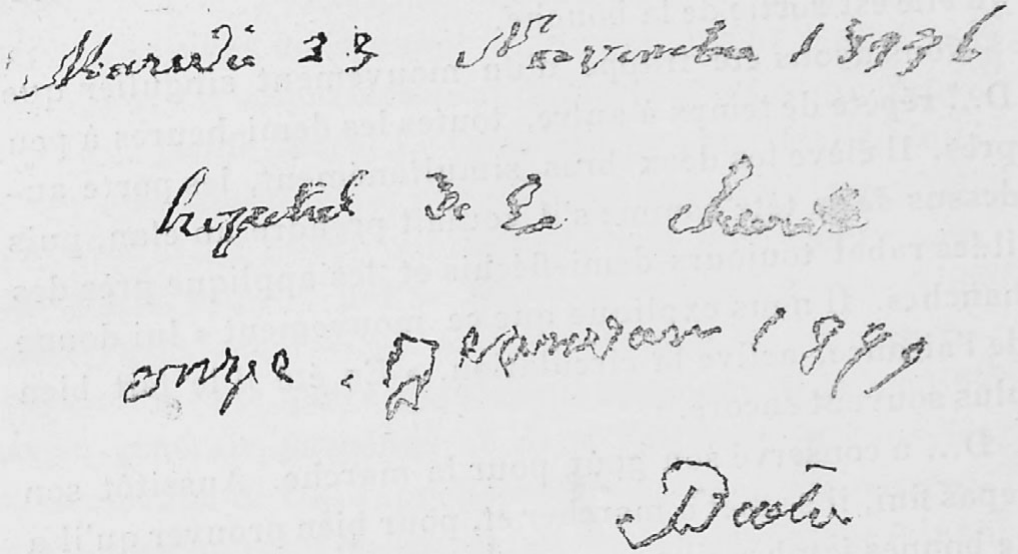
A sample of handwriting from Pierre D. included in Paul de Saint‐Legers’ thesis.

It is of interest that no alteration in cognition was observed (“*L*'*intelligence semble n*'*avoir pas varié*”) during the 3‐year period during which Pierre D. was examined. This concurred with the original description of the shaking palsy by Parkinson, although it is in contrast to recent research that has highlighted cognitive dysfunction as a common feature of late‐stage Parkinson's disease.^6^

A number of aspects of the clinical assessment would be unfamiliar to our current understanding of Parkinson's disease, for example, Saint‐Leger reported that the patient improved markedly between being seen in 1876 and 1877. As noted previously, the age at onset of symptoms would be consistent with a diagnosis of young‐onset Parkinson's disease in the present day.

Saint‐Leger concluded his report by noting that the most prominent symptom was the bearing/posture of Pierre D.—an observation that is conspicuous in the photographs—followed by his tremor (“*Après l*'*attitude*, *le phénomène le plus important est certainement le tremblement*”). The thesis then went on to consider the natural history of Parkinson's disease, reporting that cases can present with asymmetric tremor: “Beginning—there are cases where the disease begins with the tremor that suddenly appears in one limb to then invade the others successively” (“*Debut*—*il est des cas où la maladie débute par le tremblement qui apparaît tout à coup dans un membre pour envahir ensuite les autres successivement*”). This reinforced the description of asymmetry in Parkinson's disease made by James Parkinson, a key component of the differential diagnosis between Parkinson's disease and other movement disorders.^7^

The clinical description of Pierre D. sits at a key juncture in the evolution of the nosology of Parkinson's disease.^8^ Six decades on from James Parkinson's original description of the shaking palsy, the collection of symptoms that were first noted by Parkinson were being formally brought together as criteria for diagnosis.^9^ It is of note that Charcot's group was not the first to use the term *Parkinson*'*s disease*, a distinction that falls to William Rutherford Sanders, an Edinburgh physician^10^, ^11^; however, much of the clinical definition of what would now be classified as Parkinson's disease was established by Charcot and his students.^12^, ^13^

The case of Pierre D. stands out as one of the earliest identifiable individuals to be diagnosed with a disorder that is recognizable to modern neurology as Parkinson's disease and certainly one of the first cases where we have photographic evidence to accompany the diagnosis. The level of detail and identification of an individual patient is in marked contrast to Parkinson's original monograph, where a number of cases were described but in passing. In this regard, it is perhaps more reminiscent of the original case reports for Auguste D., the index case for Alzheimer's‐type dementia.^14^, ^15^, ^16^ The way in which we classify and define Parkinson's disease is currently undergoing a transformation driven by changing perceptions of nonmotor symptoms, genetics, and pathology.^17^ As such, it is instructive to return to one of the cases that helped to establish Parkinson's disease as a clinical entity as well as illuminating the human story behind one of the most iconic images in neurology.

## Author Roles

(1) Research project: A. Conception, B. Organization, C. Execution, D. Intellectual Input; (2) Manuscript: A. Writing of the first draft, B. Review and Critique.

P.A.L.: 1A, 2A, 2B

H.P.F.: 1D, 2B

M.R.: 1D, 2B

J.S.: 1D, 2B

## Full financial disclosure for the previous 12 months

## Supporting information


**Appendix** **S1.** Supporting Information.

**Supplemental figure 1** ‐ High resolution images of the original drawings of Pierre D. prepared William Gowers.

**Supplemental figure 2** – High resolution images of the original photographic prints included in Paul de Saint‐Legers thesis.
