# Supplementary material for: Pierre D. and the first photographs of Parkinson's disease
Source: Mov Disord. 2020 Jan 24;35(3):389–91. doi: 10.1002/mds.27965 (PMC7155099; doi:10.1002/mds.27965)
Supplement: Supplementary file 1 — Appendix S1. Supporting Information. [file MDS-35-389-s001.docx]

Supplemental data 1

Description of Pierre D. from Paul de Saint-Legers thesis in French and English.

CHAPITRE V.

SYMPTOMES—MARCHE—DUREE—TERMINAISONS—PRONOSTIC

Le symptôme dominant et en quelque sorte pathognomonique de la maladie de Parkinson, c’est l’attitude qui frappe bien plus encore que le tremblement, lorsqu’on observe le malade avec attention. Quand on a bien vu un de ces malheureux à l’aspect pétrifié, à l’air insouciant de tout ce qui se passe autour d’eux, se mouvant péniblement ettout d’une pièce, il devient difficile de se tromper dans le diagnostic.

On se souvient d’un acteur très-connu, aujourd’hui disparu de la scène, et qui était atteint de paralysie agitante. Si elle lui a bientôt rendu inabordables les rôles qui nécessitaient de la souplesse et de la mobilité d’expression, sa maladie lui a du moins valu en quelque sorte un sur- croît de succès dans certain rôle d: gentilhomme superbe et raide dans ses allures comme dans ses idées.

Il nous a paru intéressant de faire reproduire deux photographies, l’une de face et l’autre de profil qui nous ont été prêtées par M. Landouzy. L’histoire du malade qu'elles représentent a été publiée avec beaucoup de details dans la thèse de M. Boucher^[[1]](#footnote-1)^. En voici les traits principaux.

OBSERVATION VII.

Émotions morales vives ; tremblement très-léger limité aux mains empalement; rigidité musculaire; besoin de déambulation très-accusé; aspect de la physionomie; diminution du tremblement; diminution de la rigidité musculaire ; amélioration des forces.

D. . . . (Pierre), 56 ans, maître maçon, né à Aubusson (Creuse) d’une bonne santé habituelle. D. . . . attribue sa maladie à une vive frayeur. Le 20 mai 1871, D. . . . avait été placé contre un mur et des fédérés le mettaient en joue quand, une alerte survenant, les insurgés s’enfuirent le laissant seul plus mort que vif. Dès la fin du même mois, il remarque que ses deux mains tremblent. Ce tremblement devient surtout appréciable dans les mouvements et dans les efforts. Cependant la sensation de faiblesse et de gêne dans les membres, qui va croissant chaque jour oblige D.. à renoncer aux travaux de maçonnerie qu’il avait commencés avant la Commune. D — se souvient que, dès le 5 juin il lui fut impossible de remuer une pièce de vin de 125 litres.

Rapidement le tremblement augmente dans les mains, en même temps raideur dans les reins et les quatre membres qui s’oppose à toute espèce de mouvements étendus.

La douleur des reins qui, au début, était moindre que celle des membres, augmente très-notablement et s’étend au dos et au cou.

En octobre, D. . . . s aperçoit qu’il ne peut plus se redres- ser complètement, que ses membres inférieurs et son corps s infléchissent, en même temps le jeu des bras devient de plus en plus difficile; bientôt enfin, D. . . . remarque que sa démarche n’est plus celle de tout le monde.

*Examen du malade debout, 1876*.

Dans la station verticale, attitude de D. . . . est tellement singulière qu elle frappe autant l’attention des malades que celle des élèves.

La tête demi-fléchie, légèrement inclinée vers l’épaule droite, regarde en avant; le tronc est incurvé en avant, les cuisses sont légèrement fléchies sur le bassin. Les coudes sont collés au corps, les avant-bras, en pronation incomplète, sont à demi fléchis sur les bras, les mains en demi-flexion sont déjetées vers le bord cubital : la situation générale des doigts est telle quelle rappelle assez bien la position de la main de l’accoucheur prêt à faire la version.

Cette attitude du malade ne se modifie pas quand il se met à marcher, il se porte tout d’une pièce plutôt qu’il ne se meut: D. . . . a l'air soudé; qu’il marche, qu’il s’assoie, qu’il soit debout, qu’il se remette à marcher ou qu’il s’ar- rête, qu’il parle ou qu’il se taise, il conserve toujours la même attitude, la même fixité générale. Son faciès est des plus caractéristiques : la peau du visage d’un blanc jaunâtre, les yeux immobiles, la bouche entr’ouverte, les traits fixes, la position immuable de la tête donnent à D. . . . un air d’étonnement béat tel que les malades jus- tement frappés et de sa démarche empalée et de sa figure placide, l’ont surnommé le saint de bois.

La fixité imposée aux divers segments des membres, pas plus que la lenteur et la difficulté des mouvements, ne dépendent d’un état de faiblesse ou de paralysie des membres; elles dépendent de raideurs musculaires dont le malade a parfaitement conscience, car il se plaint constamment de sensations douloureuses le long des mémbres, sensations qu’il compare tantôt à des piqûres, tantôt à des crampes.

La difficulté et la raideur des membres existent aussi bien dans les mouvements provoqués que dans les mouvements spontanés; aussi, ce dont le malade se plaint le plus amèrement, ce n’est pas de la faiblesse, c'est de la dureté, de la raideur de ses membres. « C’est moins la force que le jeu des membres qu’on doit lui rendre. » Aussi, ajoute-t-’il avec une sorte de satisfaction mêlée d incrédulité, « il y a des médecins qui m’ont dit ‘paralysé agitant (sic), je ne suis pas paralysé, je suis fort, aussi fort que j’étais, mais les jointures sont raides. ».

Les sensations douloureuses que perçoit D. . . . sous formes de piqûres et de crampes sont plus accusées dans le décubitus assis ou dans le lit que dans la station verticale, dans la marche surtout.

D….. se plaint aussi d’avoir toujours trop chaud : la sensation de chaleur est principalement désagréable au lit, aussi le malade est-il à peine couvert et cherche-t-il à rester levé le plus longtemps possible.

Levé, jamais D... ne reste en place, depuis 5 heures du matin jusqu’à 8 heures du soir, il trotte menu sans cesse ni trêve : du matin au soir, le corps légèrement infléchi, « le nez au vent, il arpente la salle à pas égaux » ; la mar- che n’a rien de précipité et peut toujours cesser à la volonté du malade. D ne court pas après son centre de gravité, il marche et marche toujours comme poussé par une main invisible ; mais il peut régler son pas, l’accélérer ou le ralentirai peut s’arrêter au commandement : un obstacle rencontré sur sa route ne le fait pas reculer, il n’y a pas chez lui de rétropulsion spontanée ou provoquée.

*Examen détaillé de la face.*

La tête est légèrement penchée sur l’épaule droite. Les plis du front, plus élevés à gauche, sont très-accentués, surtout au niveau et au-des-sous des sourcils, qui sont relevés comme dans l’expression de l’étonnement. Les paupières se meuvent plus lente- ment que chez les personnes saines; l’occlusion prolongée des yeux se fait complètement sans efforts, mais les paupières supérieures sont animées de petits mouvements convulsifs, les yeux sont peu ouverts. Les globes oculaires regardent directement en avant; il n’y a pas de nystagmus ni de diplopie, l’acuité de la vision estnormale, les pupilles sont égales des deux côtés et resserrées, elles se contrac- tent également. L’œil du côté droit est un peu plus saillant que son congénère; les plis sont plus accentués en dehors de son petit angle qu’au côté externe de l’œil symétrique.

La lèvre inférieure, légèrement saillante en avant, est largement étalée à gauche, la commissure du même côté est fortement abaissée. La gouttière sous-nasale empiétant à droite sur la ligne médiane qu’elle dépasse, il s’ensuit que le milieu de la lèvre inférieure, déjetée elle-même dans le sens opposé, est déplacée à gauche du tubercule saillant de la lèvre supérieure

La bouche n’est pas fermée, son ouverture a la forme d’un ovoïde dont la grosse extrémité qui est à gauche mesure environ un demi-centimètre ; une ligne qui continuerait la direction de la bouche rencontrerait la ligne des yeux au niveau du Tragus droit.

Malgré cette déviation de la bouche à gauche, le sillon jugo-labial est plus profondément creusé à droite. Les deux lèvres sont immobiles. Le malade n’ouvre qu’imparfaitement la bouche, la fatigue l’empêche de la maintenir complètement ouverte plus d’une minute.

La langue est immobile quand elle est dans la cavité buccale; allongée, elle est animée d’un tremblement géné- ral, irrégulier d’abord et plus accentué dans le sens antéro- postérieur, puis uniforme. Son aspect est normal. Le nom- bre des dents est égal en haut, en bas et de chaque côté. Les lacunes formées par la chute de quelques-unes d’entre elles, égales et symétriques, ne doivent pas entrer en ligne de compte pour expliquer l’asymétrie de la face.

Une salive visqueuse s’écoule par intermittence et en faible quantité; elle s’accumule préalablement dans le vestibule de la bouche et surtout dans sa partie droite. Le sillon mento-labial est remplacé à droite par un léger relief. Les joues sont moins dépressibles que dans l’état ordinaire, elles sont inégalement déprimées, la gauche est plus creuse que la droite; elles accusent dans toute leur étendue, plus qu’à l’état habituel, les plans osseux et musculaires sous-jacents ; en haut et en arrière la sallie de la pommette, en arrière la masse quadrilatère du maxillaire, sont plus apparents que dans la norme.

Les traits de la face sont immobiles, sa partie supérieure et la région frontale expriment l’étonnement; sa partie inférieure rappelle la physionomie d’une personne qui boude.

L’intelligence semble n’avoir pas varié.

L’embarras de la parole n’a pas augmenté depuis six ans. Quand le malade veut parler, sa lèvre supérieure se relève et reste immobile; il semble hésiter; l’articulation des premières syllables est tremblée; elles se suivent lentement, elles appartiennent au registre inférieur; puis le malade hausse le ton, accélère les sons qu’il émet, ainsi que leur succession; sa voix devient en même temps plus ferme. Enfin son débit et le timbre des sons restent seuls altérés. Il semble parler entre ses dents ; la monotonie de ses phrases a quelque analogie avec une lecture faite par un enfant.

*Etat du malade en janvier 1877*.

Son état s’est singulièrement amélioré depuis son entrée a l’hôpital ; l’amélioration s’est faite lentement, insensiblement, mais l’état actuel est, sur bien des points, complètement différent de ce qu’il était en décembre 1876.

Nous allons passer successivement en revue les modifications survenues dans le tremblement, la raideur et l’accomplissement des mouvements.

*Tremblement.* — Ce symptôme est tellement affaibli, que D….. répète à qui veut l’entendre qu’il n’attend plus, pour quitter l’hôpital, que la facilité d’exécuter les mouve- ments, que la facilité de faire mouvoir ses membres les uns sur les autres.

Quand D….. est assis, les mains sur ses genoux , il faut yregarder de très-près pour voir que les mains sont animées d’un mouvement de trémulation verticale: il y a singulièrement loin de cette trémulation si peu indiquée, qu’il faut la rechercher avec soin, à l’agitation qui faisait décrire aux mains des courbes elliptiques.

Les mains étendues et les doigts écartés, on perçoit dans les phalanges un léger tremblement vertical.

Le buste est animé d’avant en arrière de petites secousses qui, malgré la faiblesse de leur intensité, ne doivent pas être rapportées, à ce qu’il nous semble, au tremble- ment imperceptible des mains. Nous croyons que le centre de ces mouvements se trouve dans les reins.

Le tremblement de la langue est sensiblement diminué; la parole est restée lente et scandée. Les autres parties du corps sont immobiles. Les émotions, les efforts, surtout si on prolonge leur durée, augmentent le tremblement des mains qui n’a jamais atteint l’intensité qu’il présentait lors de l’entrée du malade à la Charité.

*Raideur des membres; mouvements*. — Les raideurs ont singulièrement diminué; l’amélioration est telle que les membres jouent maintenant les uns sur les autres; la ra- pidité ou l’amplitude des mouvements sont assurément loin de la normale, mais le malade remue la tête, plie les reins, fléchit et étend en partie les membres, toutes choses qui étaient absolument impossibles et qui permettent de dire que D... *se désoude*.

Lors de son entrée, D... était dans un état qui offrait quelques analogies avec le début d’une rigidité cadavérique; il présentait un durcissement des muscles, une dimi- nution dans leur extensibilité et un léger raccourcissement de chacun d’eux. Aujourd’hui bon nombre de muscles sont bien moins rigides, les divers segments des membres s’étendent plus vite, plus facilement et plus amplement.

C’est surtout aux mains que l’amélioration est appréciable. Autrefois D... éprouvait une difficulté presque insurmontable pour ouvrir la main et étendre les doigts, il était forcé de prendre son temps et de faire effort; aujourd’hui il accomplit ces mouvement sans hésitation et dans un espace de temps relativement court.

Les segments des membres sont moins fléchis, la flexion est vaincue par une force moindre qu’autrefois; d’autre part, elle se reproduit moins vite, la raideur des reins di- minue. La tête, lors de l’entrée à l’hôpital, était dans une situation absolument fixe; ce n’était qu’avec une peine et une fatigue extrêmes que D... pouvait lui faire exécuter une faible partie des mouvements normaux; aujourd’hui, l’étendue du mouvement de rotation dépasse certainement 40° de chaque côté ; il y a une légère différence d’amplitude en faveur du côté gauche. Nous nous sommes arrêté pour établir la limite extrême de ces mouvements, au moment où leur accomplissement se faisait avec difficulté.

La position habituelle de la tête est toujours une demi- flexion avec inclinaison légère, latérale à droite.

Si le malade recouvre plus d’amplitude et plus de facilité dans ses mouvements, si, pour employer une expression vulgaire qui correspond bien à la réalité des choses, il se désoude, le même besoin de déambulation persiste. D... continue, comme par le passé, à circuler dans la salle tard et matin; certes son attitude générale est encore assez typique pour qu’on puisse, aujourd’hui comme autrefois, diagnostiquer d’emblée une maladie de Parkinson ; pourtant, la nécessité, la fatalité de sa marche est le fait qui pourrait frapper le plus, maintenant qu’il ne se meut plus d’une seule pièce. Ce besoin incessant de marcher est ce qui frappe le plus de prime abord. Le caractère presque fatal de cette promenade continue n’a pas échappé aux malades qui vivent avec D... ; ils lui ont donné le nom d’un personnage légendaire, fameux par sa course sans trêve à travers de nombreuses générations. Ce surnom que D... a reçu récemment mérite d’être noté, car il témoigne de la modification générale survenue et dans l’attitude et dans les mouvements. Si D... rappelle encore aujourd’hui par cer- tains côtés un saint de bois, il fait songer davantage à une statue articulée dont les membres peuvent être animés de mouvements.

La marche n’est plus la même, l’amplitude des pas a certainement augmenté. D... peut courir et tourne sur ses talons beaucoup mieux qu’il ne le faisait il y a quatre mois ; lorsqu’il le veut enfin il n’arrondit plus les angles.

Quand D... marche, il trotte menu comme autrefois, maison n’entend plus le frottement que produisait sespieds en glissant sur le parquet.

*D... est entré à Bicëtre, salle Saint-Benjamin en mai 1877, nous sommes allé le voir pour la première fois le 11 janvier 1879 (P. S.).*

Pendant ces deux dernières années, D... n’a suivi aucun traitement; à peine prend-il de loin en loin quelques bains sulfureux. Néanmoins il trouve que son état est sensibleblement amélioré ; son tremblement n’est plus apparent que dans l’exécution des mouvements. Nous sommes entré dans la salle au moment de son repas. 11 mange avec une lenteur remarquable et un air d’indifférence absolue, mais il porte franchement et tout droit son verre à la bouche et boit sans trembler. La langue ne tremble plus, même lors- qu’elle est sortie de la bouche.

Nous avons été frappé d’un mouvement singulier que D... répète de temps à autre, toutes les demi-heures à peu près. Il élève les deux bras simultanément, les porte audessus de sa tête comme s’il voulait prendre un élan, puis il les rabat toujours demi-fléchis et les applique près des hanches. Il nous explique que ce mouvement « lui donne de l’aisance, active la circulation. » En été il le fait bien plus souvent encore.

D... a conservé son goût pour la marche. Aussitôt son repas fini, il se met à marcher et, pour bien prouver qu’il a de bonnes jambes, il se met à courir jusqu’à ce que nous l’arrêtions, mais il conserve toujours une certaine raideur qui lui donne un aspect étrange. Il semble que ses bras soient ankylosés et, lorsqu’il court, il ressemble assez à un pantindont les jambesseuless’agiteraient, le reste du corps restant soudé.

Depuis un an, D... dort bien mieux ; il n’a plus la sensation de chaleur d’autrefois ; il est au contraire très-sensible au froid. Il ne souffre plus en aucune façon, sauf quelques douleurs légères qu’il ressent parfois dans les épaules de- puis les grands froids.

La salivation existe toujours, mais moins abondante.

M. le Dr Landouzy a conservé deux lignes de son écriture du 23 novembre 1876. Il nous a paru intéressant de les reproduire en les rapprochant de deux lignes écrites le 11 janvier 1879 ; on verra qu’il existe une différence, sinon très-prononcée, au moins sensible en faveur de la dernière:

Après l’attitude, le phénomène le plus important est certainement le tremblement. Mais il a ici des caracl ères particuliers sur lesquels nous reviendrons plus loin. Sui- vons d’abord par ordre les différentes phases de la maladie.

*DEBUT.*

Il est des cas où la maladie débute par le tremblement qui apparaît tout à coup dans un membre pour envahir ensuite les autres successivement. En voici unexemple très-intéressant.

CHAPTER V.

SYMPTOMS-ON-TIME-TERMINATION-PROGNOSIS

The dominant and somewhat pathognomonic symptom of Parkinson's disease is the attitude that strikes even more than the tremor, when one observes the patient attentively. When we have seen one of those unhappy people with a petrified look, with a carefree air of all that is going on around them, moving painfully and all in one piece, it becomes difficult to make a mistake in the diagnosis.

We remember a well-known actor, who had disappeared from the scene today, and who had agitated paralysis. If she soon rendered unaffordable the roles which required flexibility and mobility of expression, her illness at least gave her a certain increase of success in a certain role of a gentleman, superb and stiff in her paces. as in his ideas.

It seemed interesting to us to reproduce two photographs, one of face and the other of profile which were lent to us by Mr. Landouzy. The story of the patient they represent has been published in great detail in M. Boucher's thesis^[[2]](#footnote-2)^. Here are the main features.

OBSERVATION VII.

Bright moral emotions; very slight tremor limited to hands impalement; muscle rigidity; need for very marked ambulation; aspect of the physiognomy; decrease in tremor decreased muscle rigidity; improving forces.

D. . . (Pierre), 56 years old, master mason, born in Aubusson (Creuse) of a usual good health. D. . . He attributes his illness to great fright. May 20, 1871, D. . . he had been placed against a wall and federates put him in play when, an alert coming, the insurgents fled leaving him alone more dead than alive. At the end of the same month, he notices that his hands are trembling. This tremor becomes especially appreciable in movements and efforts. However, the feeling of weakness and embarrassment in the limbs, which grows daily, forces D. to renounce the masonry work he had begun before the Commune. D - remembers that, as of June 5, it was impossible for him to stir a piece of 125-liter wine.

Quickly the trembling increases in the hands, at the same time stiffness in the kidneys and the four limbs, which opposes any kind of extended movements.

The pain of the kidneys, which at first was less than that of the limbs, increases remarkably, and extends to the back and the neck.

In October, D.. . . He sees that he can no longer fully recover, that his lower limbs and his body are bent, at the same time the play of arms becomes more and more difficult finally, D. . . note that his approach is no longer that of everyone.

Examination of the standing patient, 1876.

In the vertical station, attitude of D.. . . It is so singular that it strikes the attention of patients as much as that of students.

The half-flexed head, slightly inclined towards the right shoulder, looks forward; the trunk is curved forward, the thighs are slightly bent on the pelvis. The elbows are glued to the body, the forearms, in incomplete pronation, are half-flexed on the arms, the half-flexed hands are deflected towards the ulnar edge: the general situation of the fingers is such that it is quite reminiscent of the position of the hand of the obstetrician ready to make the version.

This attitude of the patient does not change when he begins to walk, he wears everything in a room rather than moving: D.. . . airs welded; whether he is walking, sitting, standing, walking, stopping, talking, or same attitude, the same general fixity. Its facies is most characteristic: the skin of the yellowish-white face, the immobile eyes, the half-opened mouth, the fixed features, the immovable position of the head give to D.. . . an air of astonishment so blatant that the sick, who are justly struck, and with his impaled gait and placid face, have dubbed him the saint of wood.

The fixity imposed on the various segments of the limbs, nor the slowness and difficulty of the movements, depend on a state of weakness or paralysis of the limbs; they depend on muscular stiffness, of which the patient is perfectly conscious, for he constantly complains of painful sensations along the walls, sensations which he sometimes compares to punctures and sometimes to cramps.

Difficulty and stiffness of the limbs exist both in the induced movements and in the spontaneous movements; therefore, what the patient complains most bitterly is not weakness, it is the hardness, the stiffness of its limbs. "It's less strength than the game of the members that we must give him back. "So," he adds, with a kind of satisfaction mingled with incredulity, "there are doctors who have told me 'paralyzed agitator (sic), I am not paralyzed, I am strong, as strong as I am. was, but the joints are stiff. ".

The painful sensations that D. perceives. . . in the form of punctures and cramps are more pronounced in the seated decubitus or in the bed than in the vertical station, especially in walking.

D ... .. also complains of always being too hot: the feeling of heat is mainly unpleasant in bed, so the patient is barely covered and seeks to stay up as long as possible.

Raised, never D ... remains in place, from 5 in the morning until 8 o'clock in the evening, he trots menu constantly or truce: from morning to evening, the body slightly inflected, "the nose to the wind, he paces the room with equal steps "; the market is not rushed and can always stop at the will of the patient. D does not run after his center of gravity, he always walks and walks as if pushed by an invisible hand; but he can adjust his pace, accelerate or slow him down, stop at the command: an obstacle encountered in his path does not make him retreat, there is no spontaneous or induced retropulsion in him.

Detailed examination of the face.

The head is slightly bent over the right shoulder. The folds of the forehead, which are higher on the left, are much accentuated, especially at the level and below the eyebrows, which are raised as in the expression of astonishment. Eyelids move more slowly than in healthy persons; the prolonged occlusion of the eyes is completely effortless, but the upper eyelids are animated by small convulsive movements, the eyes are not very open. Eyeballs look directly forward; there is no nystagmus or diplopia, the acuity of vision is normal, the pupils are equal on both sides and constricted, they are also contrac- ted. The eye on the right side is a little more salient than its congeners; the folds are more accentuated outside its small angle than on the outer side of the symmetrical eye.

The lower lip, slightly protruding forward, is widely spread on the left, the commissure on the same side is strongly lowered. The sub nasal gutter encroaching on the right side of the median line that it passes, it follows that the middle of the lower lip, which is itself deflected in the opposite direction, is displaced to the left of the projecting tubercle of the upper lip.

The mouth is not closed, its opening has the shape of an ovoid whose big end which is on the left measures about half a centimeter; a line that would continue the direction of the mouth would meet the line of eyes at the level of the right Tragus.

Despite this deviation from the mouth to the left, the jugo-labial groove is deeper to the right. Both lips are motionless. The patient opens his mouth imperfectly, fatigue prevents him from keeping it fully open for more than a minute.

The tongue is still when it is in the oral cavity; elongated, it is animated by a general tremor, irregular at first, and more accentuated in the anteroposterior and then uniform direction. Its appearance is normal. The number of teeth is equal at the top, bottom and on each side. The gaps formed by the fall of some of them, equal and symmetrical, must not be taken into account to explain the asymmetry of the face.

A viscous saliva flows intermittently and in small quantities; it accumulates beforehand in the vestibule of the mouth and especially in its right part. The mento-labial sulcus is replaced on the right by a slight relief. The cheeks are less depressible than in the ordinary state, they are unequally depressed, the left is hollower than the right; they show, in all their extent, more than in the usual state, the underlying bony and muscular planes; above and behind the sallie of the cheekbone, behind the quadrilateral mass of the maxillary, are more apparent than in the norm.

The features of the face are motionless, its upper part and the frontal region express astonishment; its lower part recalls the physiognomy of a person who sulks.

The intelligence seems to have not changed.

The embarrassment of speech has not increased for six years. When the patient wants to speak, his upper lip is raised and remains motionless; he seems to hesitate; the articulation of the first syllables is trembling; they follow each other slowly, they belong to the lower register; then the patient raises his voice, accelerates the sounds he emits, as well as their succession; his voice becomes at the same time firmer. Finally its flow and the timbre of the sounds remain only altered. He seems to be talking between his teeth; the monotony of his sentences has some analogy with a reading made by a child.

State of the patient in January 1877.

His condition has improved remarkably since he entered the hospital; the improvement has been slow, imperceptibly, but the present state is, in many respects, completely different from what it was in December, 1876.

We will successively review the changes that occurred in the tremor, the stiffness and the completion of the movements.

Tremor. - This symptom is so weakened, that D ... .. repeats to anyone who will listen that he no longer expects, to leave the hospital, the ease of executing the movements, that the ease of moving its members on top of each other.

When D ... .. sits with his hands on his knees, you have to look very closely to see that the hands are animated by a vertical tremulation movement: there is singularly far from this tremulation so little indicated, that it is necessary to look for it carefully, to the agitation which made the hands describe elliptical curves.

With the hands extended and the fingers apart, a slight vertical tremor is perceived in the phalanges.

The bust is animated back and forth by little tremors which, in spite of the weakness of their intensity, must not be brought back, as it seems to us, to the imperceptible trembling of the hands. We believe that the center of these movements is in the kidneys.

The trembling of the tongue is noticeably diminished; the speech remained slow and punctuated. The other parts of the body are motionless. The emotions, the efforts, especially if one prolongs their duration, increase the trembling of the hands which never reached the intensity which it presented at the time of the entry to the Charity.

Stiffness of the limbs; movements. The stiffness has singularly diminished; the improvement is such that the members now play on each other; the swiftness or range of movement is certainly far from normal, but the patient shakes his head, bends his loins, flexes, and partially expands the limbs, all of which were absolutely impossible, and which makes it possible to say that D. .. is falling apart.

When he entered, D ... was in a state that offered some analogies with the beginning of a cadaverous rigidity; it showed a hardening of the muscles, a diminution in their extensibility, and a slight shortening of each of them. Today many muscles are much less rigid, the various segments of the limbs extend faster, easier and more amply.

It is especially in the hands that the improvement is appreciable. Formerly D ... felt an almost insurmountable difficulty in opening his hand and spreading his fingers, he was forced to take his time and make an effort; today he accomplishes these movements without hesitation and in a relatively short space of time.

The segments of the limbs are less flexed, the flexion is conquered by a lesser force than formerly; on the other hand, it reproduces less quickly, the stiffness of the kidneys decreases. The head, when entering the hospital, was in an absolutely fixed situation; it was only with extreme pain and fatigue that D could cause him to execute a small part of the normal movements; today, the extent of the rotational movement certainly exceeds 40 ° on each side; there is a slight difference in amplitude in favor of the left side. We stopped to establish the extreme limit of these movements, at the moment when their accomplishment was done with difficulty.

The usual position of the head is always a half flexion with slight inclination, lateral right.

If the patient regains more amplitude and more ease in his movements, if, to use a vulgar expression which corresponds to the reality of things, he is deserted, the same need for ambulation persists. D ... continues, as in the past, to circulate in the room late and morning; certainly his general attitude is still quite typical so that we can, today as formerly, diagnose from the start a Parkinson's disease; nevertheless, the necessity, the fatality of its march, is the fact that could strike the most, now that it is no longer moving in one piece. This incessant need to walk is what strikes the most at first sight. The almost fatal character of this continuous walk did not escape the patients who live with D ...; they gave him the name of a legendary character, famous for his unending tragedy through many generations. This nickname that D ... received recently deserves to be noted, because it testifies to the general change occurred and in the attitude and in the movements. If D ... still today recalls in some ways a wooden saint, he makes one think more of an articulated statue whose limbs can be animated by movements.

The walk is no longer the same, the range of steps has certainly increased. D ... can run and turn on his heels much better than he did four months ago; when he finally wants it he no longer rounds the angles.

When D ... walks, he trots as before, house no longer hear the friction that produced his feet sliding on the floor.

D ... entered Bicetre, room Saint-Benjamin in May 1877, we went to see it for the first time January 11, 1879 (P. S.).

During the last two years, D ... has not been treated; scarcely does he take a long distance in the distance of some sulphurous baths. Nevertheless he finds that his condition is sensibly improved; his trembling is more apparent than in the execution of movements. We entered the room at the time of his meal. He eats with remarkable slowness and an air of absolute indifference, but he carries his drink straight and straight, and drinks without trembling. The tongue does not shake, even when it is out of the mouth.

We were struck by a singular movement that D ... repeats from time to time, every half hour or so. He raises both arms simultaneously, carries them over his head as if he wanted to take a swing, then he always folds them half-bent and applies them near the hips. He explains that this movement "gives him ease, activates the circulation. In the summer he does it much more often.

D ... has kept his taste for walking. As soon as his meal is over, he begins to walk and, to prove that he has good legs, he starts to run until we stop him, but he still retains a certain stiffness that gives him an appearance strange. It seems that his arms are stiff and when he runs, he looks pretty like a panther the legsessless would agitate, the rest of the body remaining welded.

For a year, D ... has slept better; he no longer has the sensation of former warmth; on the contrary, he is very sensitive to the cold. He does not suffer in any way, except for some slight pains that he sometimes feels in the shoulders since the cold weather.

Salivation still exists, but less abundant.

Dr. Landouzy has preserved two lines of his writing of November 23, 1876. It seemed interesting to reproduce them by bringing them closer to two lines written on January 11, 1879; we will see that there is a difference, if not very pronounced, at least noticeable in favor of the latter:

After the attitude, the most important phenomenon is certainly the tremor. But here he has particular characteristics, to which we will return later. Let us first by order the different phases of the disease.

START.

There are cases where the disease begins with the tremor that suddenly appears in one limb and then invades the others successively. Here is a very interesting example.

1. Boucher. Th. inaug., 1877. [↑](#footnote-ref-1)
2. Boucher. Th. inaug., 1877. [↑](#footnote-ref-2)
