# Supplementary figures and images for: Pierre D. and the first photographs of Parkinson's disease
Source: Mov Disord. 2020 Jan 24;35(3):389–91. doi: 10.1002/mds.27965 (PMC7155099; doi:10.1002/mds.27965)

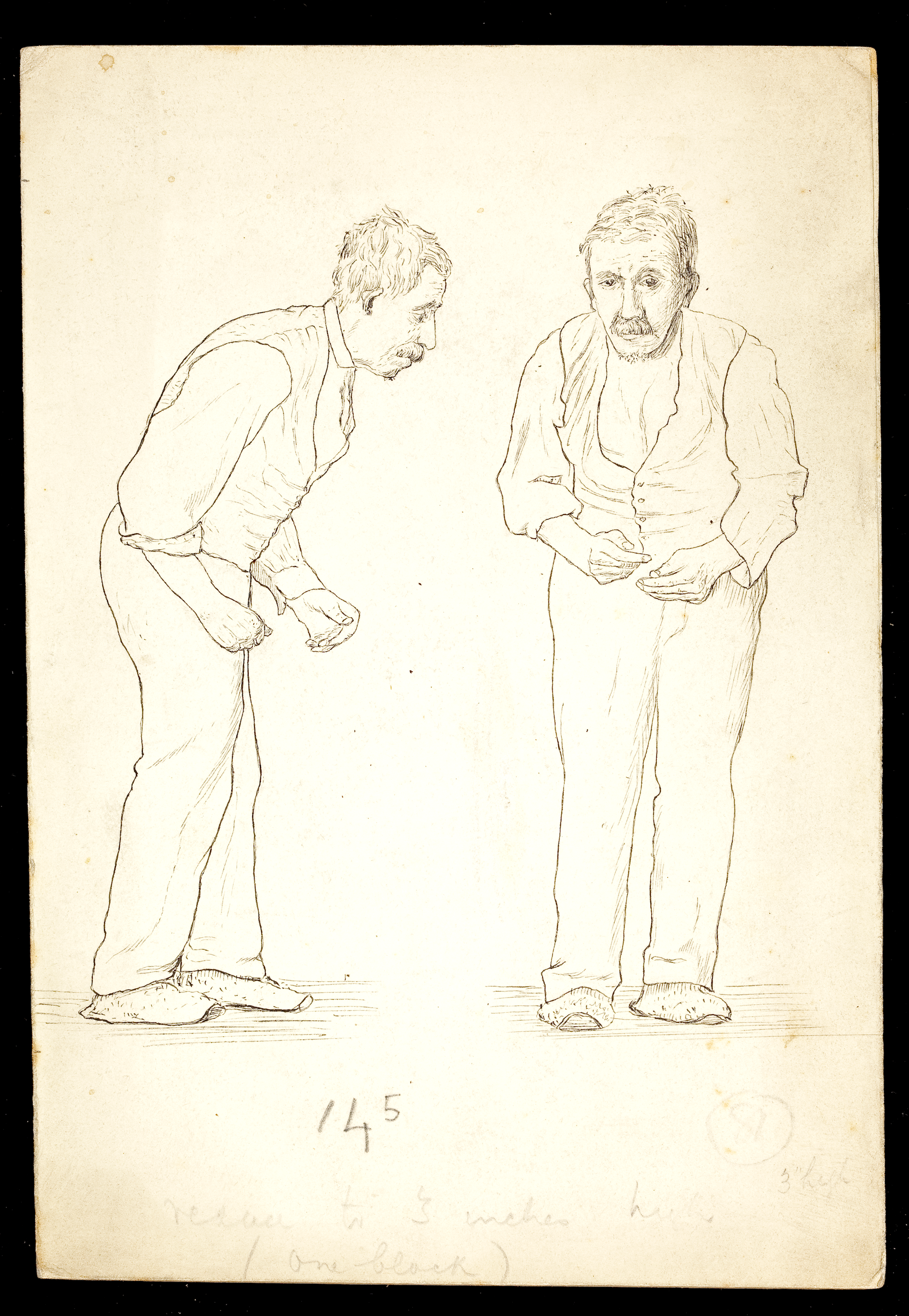

Supplement: Supplementary file 2 — Supplemental figure 1 ‐ High resolution images of the original drawings of Pierre D. prepared William Gowers. [file MDS-35-389-s002.png]

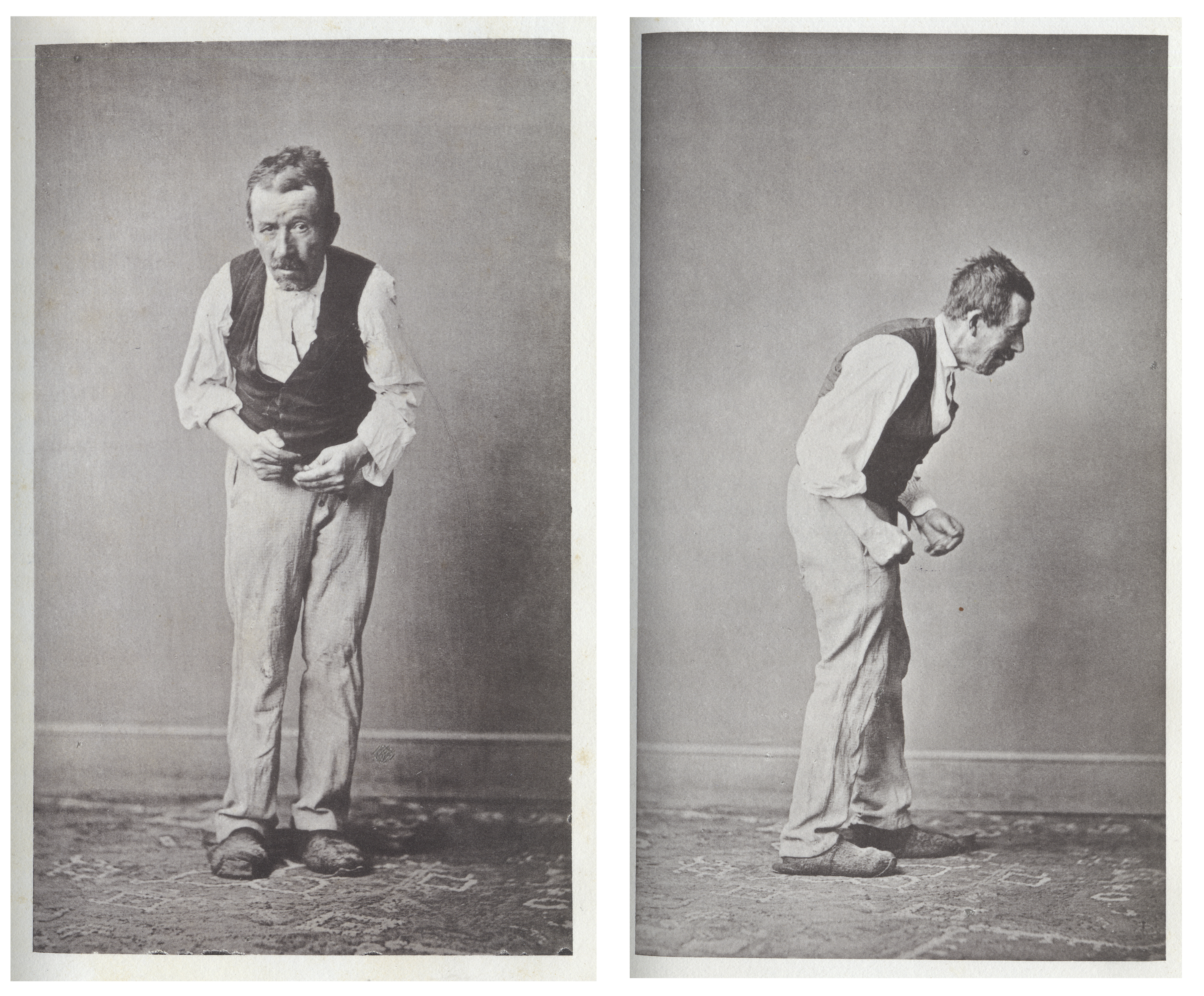

Supplement: Supplementary file 3 — Supplemental figure 2 – High resolution images of the original photographic prints included in Paul de Saint‐Legers thesis. [file MDS-35-389-s003.png]
